# Effects of elevated temperature and *p*CO_2_ on the respiration, biomineralization and photophysiology of the giant clam *Tridacna maxima*

**DOI:** 10.1093/conphys/coab041

**Published:** 2021-06-16

**Authors:** Chloé Brahmi, Leila Chapron, Gilles Le Moullac, Claude Soyez, Benoît Beliaeff, Claire E Lazareth, Nabila Gaertner-Mazouni, Jeremie Vidal-Dupiol

**Affiliations:** 1 Univ. Polynésie française, IFREMER, ILM, IRD, EIO UMR 241, F-98702 Faa’a, Tahiti, Polynésie française; 2 School of Earth Sciences, The Ohio State University, Columbus, OH 43210, USA; 3 IFREMER, IRD, Institut Louis-Malardé, Univ. Polynésie française, EIO, F-98719 Taravao, Tahiti, Polynésie française, France; 4 Laboratoire de Biologie des Organismes et Ecosystèmes Aquatiques (BOREA) MNHN, CNRS, IRD, SU, UCN, UA, Muséum National d'Histoire Naturelle, 61 Rue Buffon, CP53, 75231, Paris Cedex 05, France; 5 IHPE, Univ. Montpellier, CNRS, Ifremer, Univ. Perpignan Via Domitia, Montpellier France

**Keywords:** Giant clams, ocean acidification, photosynthetic yield, respiration, symbionts, thermal stress

## Abstract

Many reef organisms, such as the giant clams, are confronted with global change effects. Abnormally high seawater temperatures can lead to mass bleaching events and subsequent mortality, while ocean acidification may impact biomineralization processes. Despite its strong ecological and socio-economic importance, its responses to these threats still need to be explored. We investigated physiological responses of 4-year-old *Tridacna maxima* to realistic levels of temperature (+1.5°C) and partial pressure of carbon dioxide (*p*CO_2_) (+800 *μ*atm of CO_2_) predicted for 2100 in French Polynesian lagoons during the warmer season. During a 65-day crossed-factorial experiment, individuals were exposed to two temperatures (29.2°C, 30.7°C) and two *p*CO_2_ (430 *μ*atm, 1212 *μ*atm) conditions. The impact of each environmental parameter and their potential synergetic effect were evaluated based on respiration, biomineralization and photophysiology. Kinetics of thermal and/or acidification stress were evaluated by performing measurements at different times of exposure (29, 41, 53, 65 days). At 30.7°C, the holobiont O_2_ production, symbiont photosynthetic yield and density were negatively impacted. High *p*CO_2_ had a significant negative effect on shell growth rate, symbiont photosynthetic yield and density. No significant differences of the shell microstructure were observed between control and experimental conditions in the first 29 days; however, modifications (i.e. less-cohesive lamellae) appeared from 41 days in all temperature and *p*CO_2_ conditions. No significant synergetic effect was found. Present thermal conditions (29.2°C) appeared to be sufficiently stressful to induce a host acclimatization response. All these observations indicate that temperature and *p*CO_2_ are both forcing variables affecting *T. maxima*’s physiology and jeopardize its survival under environmental conditions predicted for the end of this century.

## Teaser

In this article, we mimicked current and future warm season conditions and showed that temperature and *p*CO_2_ predicted for the end of this century are forcing variables significantly affecting *Tridacna maxima*’s growth and photophysiology in an order of magnitude that will jeopardize its survival in French Polynesian lagoon.

## Introduction

Anthropocene era is characterized by human activities releasing gigatons of CO_2_ in the atmosphere and contributing to induce global climate change (Broeker *et al.*, 1979; Caldeira and Wickett, 2003; Sabine *et al.*, 2004; Zeebe *et al.*, 2008; IPCC, 2014). The transfer of CO_2_ from the atmosphere to the ocean results consequently to the increase of dissolved CO_2_ in the seawater inducing significant pH decreases. On the other hand, due to the ‘greenhouse gas’ property of CO_2_, terrestrial and sea surface temperature rise constantly (i.e. global warming). According to the last Intergovernmental Panel on Climate Change report (IPCC, 2019), warming had already reached +1°C by comparison to the pre-industrial period and may reach +1.5°C around 2040 if the current rate of emission is maintained. Enrichment of dissolved CO_2_ in the ocean modifies the carbonate chemistry by decreasing carbonate ion concentration ([CO_3_^2−^]) and releasing protons that respectively decrease calcium carbonate saturation state (Ω) and seawater pH, i.e. ocean acidification (Kleypas *et al.*, 1999; Caldeira and Wickett, 2003). Indeed, pH had already decreased by 0.1 pH unit and this drop may reach −0.3 by 2100 (according to the RCP (Representative Concentration Pathway) 8.5 scenario; IPCC, 2019).

CO_3_^2−^ is a carbonate ion form involved in biologically controlled calcification process, i.e. biomineralization (Orr *et al.*, 2005) of many marine organisms such as scleractinian corals and molluscs constructing calcium carbonate structures (e.g. exoskeleton, shell, test, spicule). This characteristic of their physiology make them highly sensitive to ocean acidification (Hoegh-Guldberg *et al.*, 2007; Ries *et al.*, 2009; Kroeker *et al.*, 2013; Gazeau *et al.*, 2013; Parker *et al.*, 2013), especially when this skeleton is made of aragonite, which is more sensitive to dissolution than calcite (Morse *et al*., 2007). Among marine calcifying molluscs (e.g. oysters, mussels, giant clams, abalones, limpets) negative impacts of temperature and *p*CO_2_ have been demonstrated on the survival, growth, biomineralization processes and other key physiological functions on different stages of their life cycle (Bougrier *et al.*, 1995; Kurihara, 2008; McClintock *et al.*, 2009; Gazeau *et al.*, 2010; Welladsen *et al.*, 2010; Melzner *et al.*, 2011; Rodolfo-Metalpa *et al.*, 2011; Schwartzmann *et al.*, 2011; Talmage and Gobler, 2011; Liu *et al.*, 2012; Watson *et al.*, 2012; Kurihara *et al.*, 2013; Fitzer *et al.*, 2014; Le Moullac *et al.*, 2016a, b; Meng *et al.*, 2018; Wessel *et al.*, 2018; Avignon *et al.*, 2020). However, effects of each stressor and their potential synergetic effect on giant clam’s physiology are still poorly understood.

Giant clams produce an aragonitic shell composed of a prismatic inner layer and a crossed-lamellar outer layer (Pätzold *et al.*, 1991; Faylona *et al.*, 2011; Agbaje *et al.*, 2017; Gannon *et al.*, 2017). These organisms form an extracellular phototrophic mutualistic symbiosis with dinoflagellates of the Symbiodiniaceae family (Holt *et al.*, 2014; LaJeunesse *et al.*, 2018). The symbionts are hosted in the mantle part exposed to the light, in a tubular system (Z-tubules) directly connected to the stomach (Norton *et al.*, 1992; Holt *et al.*, 2014). The resulting mixotrophic organisms therefore acquire nutrients from heterotrophic (seawater filtration) and photo-autotrophic pathways (Klumpp *et al.*, 1992; Hawkins and Klumpp, 1995). Nutrients provided by symbionts (such as glucose; Ishikura *et al.*, 1999) may account for a major part of the giant clam’s energy needs (Klumpp *et al.*, 1992; Klumpp and Griffiths, 1994; Klumpp and Lucas, 1994; Hawkins and Klumpp, 1995; Elfwing *et al.*, 2002; Yau and Fan, 2012; Holt *et al.*, 2014; Soo and Todd, 2014).

Despite their ecological and socio-economic importance, effects of thermal stress and acidification on giant clams still need to be investigated. Thermal stress has shown to decrease fertilization success in *T. maxima* (Armstrong *et al.*, 2020), decrease oxygen production in *Tridacna derasa* and *Tridacna gigas* as well as respiration rates in *T. derasa* species (Blidberg *et al.*, 2000). In contrast, an increase of respiration with a high photosynthetic rate in the holobiont was reported for *Tridacna squamosa* (Elfwing *et al.*, 2001). Thermal stress also reduces the abundance of symbionts in *T. gigas* (Leggat *et al.*, 2003) and *Tridacna crocea* (Zhou *et al.*, 2019) and a decrease of photosynthate export from symbionts to the host (Leggat *et al.*, 2003). In *T. maxima*, heat stress was shown to induce changes in the fatty acid composition, lipid pathways and the overexpression of genes encoding reactive oxygen species (ROS) scavengers (Dubousquet *et al.*, 2016). In symbionts hosted in *T. gigas*, high-light levels and heat stress caused a decrease of symbiont density, cell size and chlorophyll content (Buck *et al.*, 2002). Toonen *et al.* (2012) demonstrated that low pH combined with high nutrient concentration have different impacts on shell growth rate depending on the *Tridacna* species. Moreover, Watson (2015) showed that high-light irradiance condition (i.e. high photosynthetically active radiation level) may limit the impact of *p*CO_2_ on shell growth and total animal mass gain. Regarding the potential synergetic effect of temperature and *p*CO_2_ parameters, the only study carried out showed that the survival of *T. squamosa* juveniles may decrease by ocean warming and acidification (Watson *et al.*, 2012). To our knowledge, no study had investigated the impacts of both parameters, and their potential synergetic effect, on several key physiological parameters of both *T. maxima* host and its symbionts. To fill this gap, our experimental approach was designed to better understand the physiological mechanisms underlying the response of giant clams to global climate changes.

In French Polynesia, *T. maxima* (Röding, 1798) is one of the most emblematic and patrimonial species. It represents an important food resource for inhabitants of remote atolls and giant clam fishery and aquaculture activities generate substantial incomes for local fishermen and farmers (Van Wynsberge *et al.*, 2016; Andréfouët *et al.*, 2017). However, wild and cultivated giant clam stocks are largely threatened by environmental disturbances such as abnormally high sea surface temperature inducing mass mortality events as reported in Tuamotu atolls by Addessi (2001) and Andréfouët *et al.* (2013). Like the symbiotic coral species, bleaching events (e.g. the symbiosis dissociation) affecting giant clam have been recorded several times in the Indo-Pacific Region (Addessi, 2001; Buck *et al.*, 2002; Leggat *et al.*, 2003; Andréfouët *et al.*, 2013, 2015; Junchompoo *et al.*, 2013) in association to seawater temperature increase by a few degrees above the seasonal maximum (Addessi, 2001; Andréfouët *et al.*, 2013). More recently, two mass bleaching events of giant clams occurring in Reao and Tatakoto (Tuamotu islands) were linked to a prolonged exposure to high temperature (≥30°C over several weeks) (Andréfouët *et al.*, 2017; see Supplementary Fig. S1).

Based on a 65-day crossed-factorial experiment, we investigated physiological responses of 4-year-old *T. maxima* to temperature and *p*CO_2_ conditions in the French Polynesian lagoons during the present warmer season and those predicted for 2100 by the IPCC 2014: +1.5°C (RCP 4.5 scenario) and +800 *μ*atm of CO_2_ (RCP 8.5 scenario). The effect of each parameter and their potential synergetic effect were evaluated based on respiration, biomineralization and photophysiology by analyzing the holobiont O_2_ production and respiration, growth rate, ultrastructure of the shell, symbiont density and photosynthetic yield. In addition, the kinetics of thermal and acidification stress were assessed by performing analyses at different time of exposure (i.e. 29, 41, 53, 65 days).

**Table 1:** Measured and calculated parameters of seawater for all treatments

Treatments	Temperature (°C)	*p*CO_2_ (*μ*atm)	pH_NBS_	Salinity (‰)	A_T_ (μmol/kg SW)	DIC (μmol/kg SW)	Ω_aragonite_
Control	29.2 (±0.1)	428 (±21)	8.19 (±0.01)	35	2422 (±170)	2071 (±85)	4.17
Thermal stress	30.7 (±0.1)	431(±25)	8.19 (±0.01)	35	2409 (±157)	2051 (±89)	4.14
Acidification stress	29.2 (±0.1)	1210 (±41)	7.81 (±0.03)	35	2466 (±90)	2306 (±34)	1.99
Acidification and thermal stress	30.7 (±0.1)	1213 (±29)	7.81 (±0.03)	35	2423 (±110)	2252 (±58)	2.02

Total alkalinity (A_T_) is given in mean (±SD) based on weekly measurements for each experimental tank and for each condition. *p*CO_2_, Ω_aragonite_ and dissolved inorganic carbon (DIC) were calculated using the CO_2_SYS software.

## Materials and methods

### Biological material

Two hundred juvenile *T. maxima* (4-year-old, ~5–6 cm height), with brownish/dark-green colour, were collected from the cultivated stock in Reao lagoon (Tuamotu islands). Then, they were exported to the Centre Ifremer du Pacifique in Tahiti where they were acclimatized in outdoor tank continuously renewed with natural, unfiltered, lagoonal seawater providing a constant input of microflora and fauna at natural concentration. Each individual was placed onto a petri dish on which byssal gland further developed for attachment. All individuals directly opened up right after their transfer into the outdoor tank. No visual sign of stress was observed during the acclimation period except for 4 individuals that died within the first 3 days (corresponding to a 2% mortality rate). Specimens used in this study were collected and held under a special permit (MEI #284) delivered by the French Polynesian government.

### Experimental design and rearing system

To study the impact of temperature, *p*CO_2_, and their synergetic effect on the physiology of the giant clams and their symbionts, 4 experimental conditions were set up by applying 2 temperatures (29.2°C and 30.7°C) and 2 levels of *p*CO_2_ (430 ± 22 *μ*atm and 1212 ± 35 *μ*atm). The tested conditions were as follows: (i) control: 29.2°C, 430 *μ*atm; (ii) acidification stress: 29.2°C, 1212 *μ*atm; (iii) thermal stress: 30.7°C, 430 *μ*atm; and (iv) acidification and thermal stress: 30.7°C, 1212 *μ*atm (Table 1). Temperature was set and maintained with an electronic controller (Hobby Biotherm Professional) connected to an aquarium heater (Shego). The pH was manipulated by bubbling CO_2_ in water tanks. This was controlled by a pH-stat system (Dennerle) that continuously monitored pH (calibrated to NIST scale) and temperature to control the quantity of CO_2_ to maintain the desired pH. The light was set to obtain a photosynthetically active radiation of 200 ± 20 μmol of photons m^−2^.s^−1^ on a 12:12 h light/dark photoperiod.

After a 3-week acclimation period in an outdoor tank, 96 clams were randomly distributed in the experimental tanks 1 week before starting the experiment. For each condition, we used a 500-l tank containing 4 tanks of 30 l (ecological replicates) renewed with natural unfiltered lagoonal seawater at a flow rate of 50 l/h providing a constant input of microflora and fauna at natural concentration. Each 30-l tank contained 6 clams (biological replicates). To avoid physiological shock, targeted temperature and *p*CO_2_ were linearly achieved over 7 days. To evaluate the kinetics of the thermal and/or acidification stress, analyses were performed at 4 different times of exposure, i.e. 29, 41, 53 and 65 days. In total, 64 clams were used for data acquisition corresponding to 4 individuals per condition (1 individual per 30-l tank) and per time of exposure.

### Monitoring of temperature, pH and water quality

To insure the stability of experimental conditions, temperature and pH parameters were measured twice a day for each tank at 8:00 am and 4:00 pm using a mercury thermometer certified ISO 9001 (±0.1°C accuracy) and a pH-meter Consort P603 (±0.01 accuracy). Total alkalinity (TA) was weekly titrated using a 0.01-N HCl solution and a titrator (Schott Titroline Easy). Levels of *p*CO_2_ and aragonite saturation state were calculated from temperature, pH (NBS scale), salinity and mean TA using the CO_2_SYS software (van Heuven *et al.*, 2009). All parameters including seawater carbonate chemistry are reported in Table 1.

### Holobiont O_2_ consumption and production measurements

Giant clams were placed in an ecophysiological measurement system (EMS) to monitor O_2_ consumption and production. The EMS consisted of five open-flow chambers. Four giant clams were individually placed into four chambers, while an empty shell was placed into a fifth chamber used as a control. EMS chambers contained water at the same temperature and *p*CO_2_ conditions as in the experimental tanks. The light energy and photoperiod conditions were the same as for the acclimation tanks. Flow rate in all chambers was constantly maintained at 12 l.h^−1^. Each chamber was equipped with a two-way electromagnetic valve activated by an automaton (FieldPoint National Instruments). When the electro-valve was opened, the water released from the chamber was analyzed for 3 min using an oxygen sensor (OXI 538, Cellox 325, WTW, Weilheim, Germany) to quantify dissolved oxygen. Oxygen measurements were performed over 48 h. The first 8 h of measurement were discarded due to the animal acclimatization to the chamber. In each chamber, the cycle was completed within 3 min: the first 2 min served to stabilize the measurement and an average of oxygen data was performed on the last minute of acquisition. This cycle was followed by another time frame of 3 min in the control chamber following the sequence specimen #1, control, specimen #2, control, specimen #3, control, specimen #4, control.

Respiration rate (RR) and production rate (PR) were calculated from the data obtained during night time and day time, respectively, using differences in oxygen concentrations between the control and experimental chambers. RR and PR = V(O1 − O2), where O1 is the oxygen concentration in the control chamber, O2 is the oxygen concentration in the experimental chamber and V is the water flow rate. RR and PR data were normalized to tissue dry weight. Once normalized, the terminology becomes O_2_ consumption for RR and O_2_ production for PR; both expressed in mg O_2_.h^−1^.g^−1^ dry weight.

After O_2_ production and O_2_ consumption analyses were completed, a piece of the mantle was dissected for further symbiont fluorescence and density analyses (see Section Fluorescence and density measurements of symbionts). The remaining soft tissues were frozen and lyophilized for RR and PR data normalization.

### Fluorescence and density measurements of symbionts

Potential effect of temperature and *p*CO_2_ conditions on the photophysiology of the symbionts was studied by comparing fluorescence yield of photosystem II (PSII) between all experimental conditions. After clams were sacrificed, a 1 × 2-cm mantle fragment was dissected. The tissue fragment was gently swiped using tissue paper to remove excess mucus and symbionts were collected by doing 5 smears using a sterilized razor blade. Collected symbionts were diluted into 5-ml of 0.2-μm filtered seawater and placed at the obscurity for 10 min to inactive the PSII before light excitation. Samples were homogenized and 3 ml of the homogenate were collected, placed into a quartz-glass cuvette and analyzed with AquaPen fluorometer (APC-100, Photon System Instruments®, Czech Republic) at a 450-nm wavelength. The minimal fluorescence (*F_0_*) and the maximal fluorescence (*F_m_*) were measured. The quantum yield of photosynthesis was calculated as *F_v_/F_m_* where *F_v_* is the variable fluorescence (*F_v_* = *F_m_*–*F_0_*).

In addition, symbiont densities were evaluated from mantle fragments. For each individual, a circular (5 mm in diameter) piece of mantle was collected using a punch. The piece was weighed, grounded in 0.2-μm filtered seawater and homogenized. Then, 20 μl of the tissue extract were immediately collected and placed into Malassez cells for symbiont counting under optical microscope. For each sample, counting was performed on 4 replicates (3 columns per replicate). Data are expressed in number of symbionts/mg of mantle tissue.

### Calcein labelling for evaluating daily shell extension rate

To study the impact of temperature and *p*CO_2_ on shell growth rate, the mineralization front of giant clams was marked using calcein fluorochrome, which is irreversibly precipitated at the CaCO_3_ mineralization site. Before the experiment starts, giant clams were immersed in a 100-mg.l^−1^ calcein solution (Sigma Aldrich) (calcein diluted in 1-μm filtered-seawater) for 8 h in the dark. During the labelling procedure, the bath of calcein solution was aerated using bubblers and water current was created via pumps. Calcein-labelled specimens were then placed into the experimental tanks. At the end of the experiment, for each individual, a 5-mm thick section was cut along the maximal shell growth axis through the right valve using a Swap Top Inland® diamond saw. All sections obtained were polished and observed under epifluorescence with a Leitz Dialux® 22 microscope. The distance between the fluorescent calcein mark and the edge of the shell formed during the experiment was measured following the maximal growth direction. Daily shell extension rate (expressed in μm.day^−1^) was obtained by dividing the measured distance by the number of days of incubation in experimental conditions.

### Shell scanning electron microscopy study

To characterize temperature and *p*CO_2_ effect on the shell ultrastructure, a scanning electron microscopy (SEM) study was carried out. For each individual, a 10-mm thick shell section was cut facing the section used for calcein observations. The section was then fractured along the width, 2 cm below the growing part of the shell (marginal part), using a small chisel and a hammer. Then, the apical fragment was longitudinally fractured and one piece was sonicated in tap water for 10 s, air dried and an additional drying was done overnight at 35°C. Sample was placed on a stub covered with carbon tape, gold-coated and observed at 15 kV using a Hitachi TM3030 SEM at the Université de la Polynésie française. For each condition, and for each time of exposure, two samples were selected based on their daily shell extension rate, i.e. samples showing the lowest and the highest rate. To evaluate the impact of temperature and/or *p*CO_2_ on the shell ultrastructure, SEM observations were performed for each specimen in two different zones of the crossed-lamellar outer layer. Zone 1 corresponds to the shell formed *in situ* (i.e. in the shell region located before the calcein mark), while zone 2 corresponds to the shell formed during the experiment. Observations were made at the ultrastructural level and focused on the aspect and integrity of the lamellae of the crossed-lamellar outer layer. In addition, to check the potential effect of temperature and *p*CO_2_ on the external shell surface, the periostracum (i.e. the organic layer covering the shell external surface) of two individuals per experimental condition (65 days of exposure) was observed.

### Statistical analyses and data processing

Normality of data distribution and homogeneity of variance were tested with the Shapiro–Wilk test and the Bartlett test, respectively. Production and consumption of O_2_ data followed the conditions of application of parametric tests, but photosynthetic yields and symbiont densities were transformed using Box Cox transformation while shell extension rates were square root transformed to meet these conditions. Comparisons were done using a three-way ANOVA with interactions (fixed factors: time of exposure, temperature and *p*CO_2_). Tukey post hoc comparisons were done at α = 0.05 for all analyses. Correlations between physiological parameters were tested using Pearson method with a threshold of *r* = 0.25 (α = 0.05).

For all physiological parameters, i.e. O_2_ respiration, O_2_ consumption, symbiont photosynthetic yield and density, means (±SD) were calculated based on the four biological replicates for each condition and each time of exposure. For the daily shell extension rate, means (±SD) were calculated for each condition and each time of exposure based on the number of biological replicates displaying a detectable calcein mark.

## Results

### Effect of temperature and pCO_2_ on the holobiont oxygen balance

**Figure 1:**
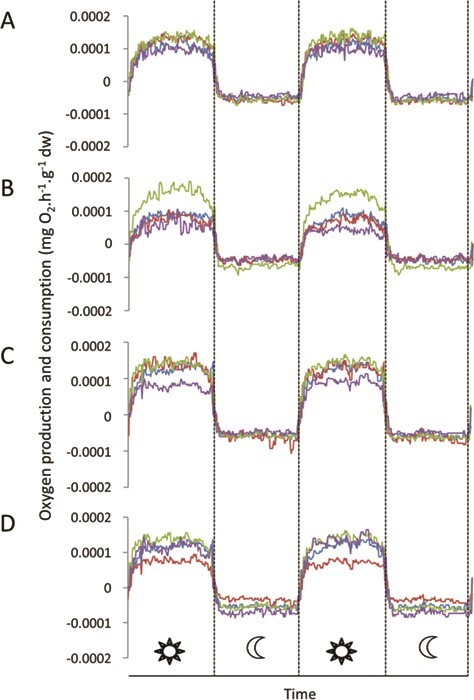
Variations of O_2_ production and O_2_ consumption acquired over a 48-h period after 29 days of exposure to different temperature/*p*CO_2_ conditions: (**A**) 29.2°C, 430 *μ*atm of CO_2_; (**B**) 30.7°C, 430 *μ*atm of CO_2_; (**C**) 29.2°C, 1212 *μ*atm of CO_2_; and (**D**) 30.7°C, 1212 *μ*atm of CO_2_. Data are expressed in mg O_2_.h^−1^.g^−1^ tissue dry weight (dw) and correspond to day-time and night-time acquisitions for four replicates per condition (each color line represents one replicate).

For all sampling time (i.e. 29, 41, 53, 65 days), a cyclic pattern of O_2_ production and consumption was observed following the circadian cycle (Fig. 1). This pattern corresponds to oxygen photosynthetically produced and heterotrophically consumed by the holobiont during the day and the night, respectively. No mortality was observed in all tested temperature/*p*CO_2_ conditions during the whole experiment.

**Figure 2:**
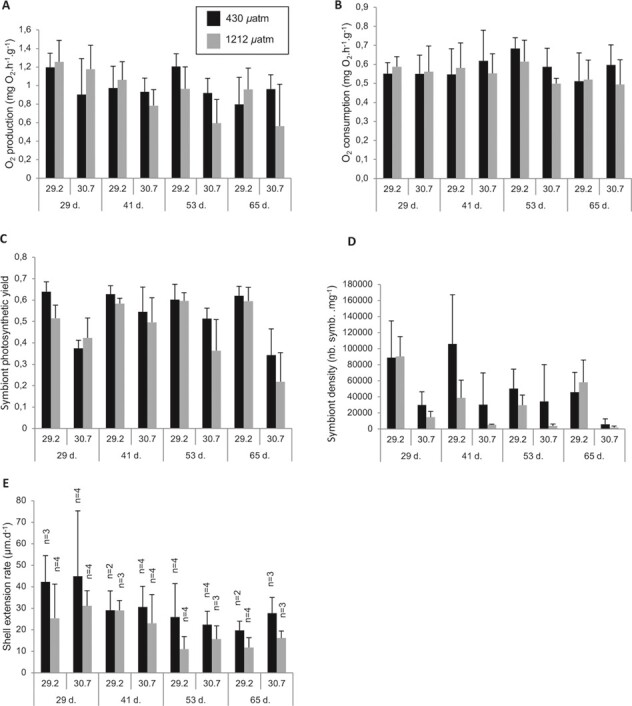
Graphs reporting data of (**A**) oxygen production, (**B**) oxygen consumption, (**C**) symbiont photosynthethic yield, (**D**) symbiont density and (**E**) daily shell extension rate obtained for each temperature/*p*CO_2_ experimental condition and time of exposure (black and grey columns correspond to 430 *μ*atm and 1212 *μ*atm, respectively). Data are given in mean (+SD) calculated from four replicates for all physiological parameters except for daily shell extension rate for which numbers of replicates are specified.

Mean values of normalized O_2_ production and consumption are shown in Fig. 2. Tukey post hoc test results are reported in Table 3. The ANOVA has indicated that oxygen production of the holobiont during day time was significantly altered at 30.7°C (*P* = 0.009) but not at 1212 *μ*atm of CO_2_ (*P* = 0.362) (Table 2). In addition, O_2_ production was higher at 29.2°C than at 30.7°C for all times of exposure (Fig. 2A) and decreased over time (*P* = 0.030, Table 2). The night-time O_2_ consumption, however, was not significantly influenced by temperature (*P* = 0.590) neither by *p*CO_2_ (*P* = 0.361) nor by time of exposure (*P* = 0.533) and remained stable throughout the whole experiment (Fig. 2B). No interaction effect between the three tested factors was found to affect the holobiont oxygen balance (Table 2).

**Table 2:** Results from the three-way ANOVA performed on holobiont O_2_ production and consumption data, symbiont photosynthetic yield, symbiont density and giant clam shell extension rate

		O_2_ production (*n* = 64)	O_2_ consumption (*n* = 64)	Photosynthetic yield (*n* = 64)	Symbiont density (*n* = 64)	Shell extension rate (*n* = 55)
Time	*F*	3.264	0.740	3.566	6.035	4.695
	*P*	0.030^*^	0.533	0.021^*^	0.001^***^	0.007^**^
Temperature	*F*	7.551	0.294	65.200	79.158	0.466
	*P*	0.009^**^	0.590	<0.0001^***^	<0.0001^***^	0.499
*p*CO_2_	*F*	0.846	0.851	6.125	8.665	7.409
	*P*	0.362	0.361	0.017^*^	0.005^**^	0.010^**^
Time × temperature	*F*	0.413	0.965	3.393	1.528	0.319
	*P*	0.744	0.417	0.025^*^	0.219	0.812
Time × *p*CO_2_	*F*	1.765	0.476	0.024	1.033	0.251
	*P*	0.167	0.700	0.995	0.386	0.860
Temperature × *p*CO_2_	*F*	1.334	1.029	0.100	1.989	0.026
	*P*	0.254	0.315	0.753	0.165	0.872
Time × temperature x *p*CO_2_	*F*	1.216	0.148	2.153	0.474	0.336
	*P*	0.314	0.930	0.106	0.702	0.799

The three fixed factors are *p*CO_2_, temperature and time of exposure.

Asterisks denote significant differences (^*^P  ≤  0.05, ^**^P  ≤  0.01, ^***^P  ≤  0.001).

**Figure 3:**
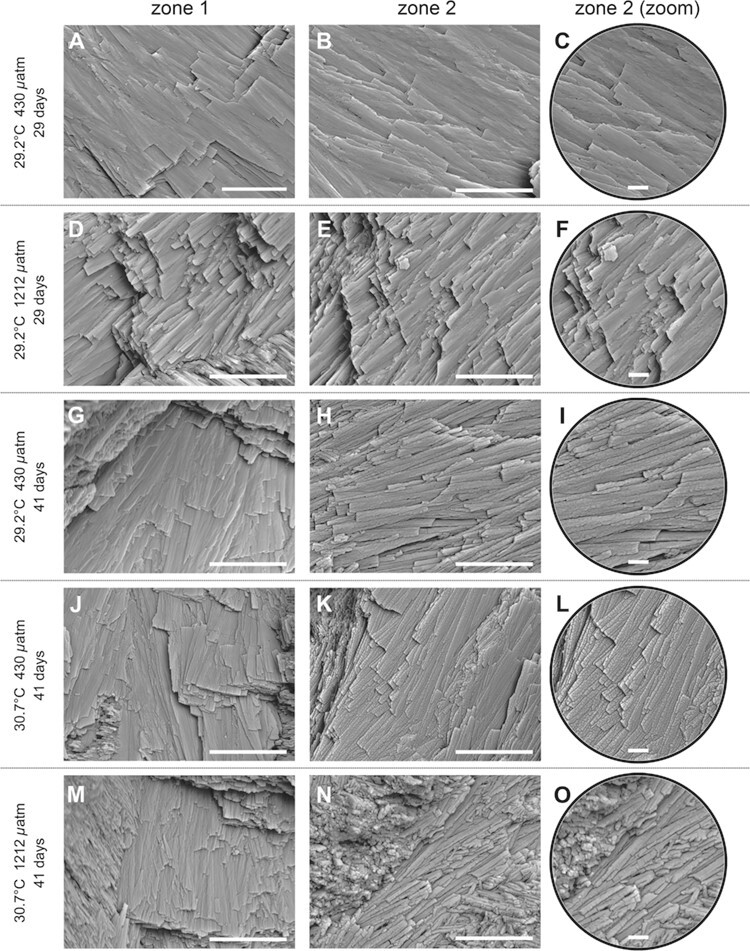
Effects of experimental conditions on the ultrastructure of the *T. maxima* shell outer layer investigated via SEM. (**A**, **B**, **D**, **E**, **G**, **H**, **J**, **K**, **M**, **N**: scale bar, 10 μm; **C**, **F**, **I**, **L**, **O**: scale bar, 2 μm). Zone 1 and zone 2 correspond to the shell formed *in situ* (i.e. before the experiment) and in the experimental conditions, respectively. At 29 days, no significant differences between zones 1 and 2 are observed (A–C: 29.2°C, 430 *μ*atm; D–F: 29.2°C, 1212 *μ*atm). In both zones, lamellae are well cohesive, displaying elongated shape with a smooth surface and slightly rounded outlines. This latter microstructural feature is observed in all shells formed *in situ* (zone 1), for all temperature/*p*CO_2_ conditions and all time of exposure (A, D, G, J, M). At 41 days, lamellae in zone 2 are less cohesive with or without pronounced granular aspect (H, I: 29.2°C, 430 *μ*atm; K, L: 30.7°C, 430 *μ*atm; N, O: 30.7°C, 1212 *μ*atm).

**Table 3:** Results from Tukey post hoc tests following the three-way ANOVA performed on analyzed physiological parameters

		O_2_ production	Photosynthetic yield	Symbiont density	Shell extension rate
Time	29 days	a	a	a	a
	41 days	ab	ab	ab	ab
	53 days	ab	ab	b	b
	65 days	b	b	b	b
Temperature	29.2 °C	a	a	a	-
	30.7 °C	b	b	b	-
*p*CO_2_	430 *μ*atm	-	a	a	a
	1212 *μ*atm	-	b	b	b
Time × temperature	29 days × 29.2°C	-	ab	-	-
	41 days × 29.2°C	-	a	-	-
	53 days × 29.2°C	-	a	-	-
	65 days × 29.2°C	-	a	-	-
	29 days × 30.7°C	-	cd	-	-
	41 days × 30.7°C	-	abc	-	-
	53 days × 30.7°C	-	bcd	-	-
	65 days × 30.7°C	-	d	-	-

The effects of significant parameters were tested as time of exposure alone and combined to the temperature.

The letter annotations correspond to the significances between conditions (*P* < 0.05).

**Table 4:** Compilation of ultrastructural observations of lamellae of the shell outer layer and the periostracum using SEM

Time of exposure	Temperature (°C)	*p*CO_2_ (*μ*atm)	Sample	Lamellae of outer shell layer	Periostracum
29 days	29.2	430	A	ND	
			B	ND	
	30.7	430	A	ND	
			B	ND	
	29.2	1212	A	ND	
			B	ND	
	30.7	1212	A	ND	
			B	ND	
41 days	29.2	430	A	D	
			B	D	
	30.7	430	A	D	
			B	D	
	29.2	1212	A	D	
			B	D	
	30.7	1212	A	D	
			B	ND	
53 days	29.2	430	A	D	
			B	D	
	30.7	430	A	D	
			B	D	
	29.2	1212	A	ND	
			B	D	
	30.7	1212	A	D	
			B	ND	
65 days	29.2	430	A	D	No alteration
			B	D	No alteration
	30.7	430	A	D	No alteration
			B	ND	No alteration
	29.2	1212	A	D	No alteration
			B	D	No alteration
	30.7	1212	A	ND	No alteration
			B	D	No alteration

‘A’ and ‘B’ represent the two samples observed for each treatment and time of exposure; ‘ND’ means that no significant difference is observed between the shell formed before and during the experiment for the lamellae of the outer shell layer; ‘D’ means that a notable difference is observed between both parts of the shell (i.e. the lamellae are less cohesive).

For the periostracum, ‘no alteration’ means that the periostracum covering the ventral extremity part of the shell is well preserved.

### Symbiont density and photosynthesis under different temperature/*p*CO_2_ conditions

After 2 months of exposure, the symbiont photosynthetic yield and density were significantly impacted at 30.7°C (*P* < 0.0001), high *p*CO_2_ (1212 *μ*atm) (*P* = 0.017 and *P* = 0.005, respectively) and by the time of exposure (*P* = 0.021 and *P* = 0.001, respectively). During the whole experiment and for both *p*CO_2_ conditions, the means of photosynthetic yield (Fig. 2C) and symbiont density (Fig. 2D) tended to be higher at 29.2°C than at 30.7°C. However, an inter-individual variability was observed.

Concerning the interaction parameters, only interaction between temperature and time of exposure parameters had a significant effect on the symbiont photosynthetic yield (*P* = 0.025) (Table 2). No parameter interaction was found to affect the symbiont density (Table 2).

### Effect of pCO_2_ on daily shell extension rate

Calcein mark was detectable in 55 over 64 shell sections. Statistical analyses on shell extension rate showed that *p*CO_2_ and the time of exposure had a significant effect on the shell extension rate (*P* = 0.010 and *P* = 0.007, respectively, Table 2). As shown in Fig. 2E, the mean values of shell extension rates tended to be lower at high *p*CO_2_.

### Effect of long-term exposure to temperature and pCO_2_ on the periostracum and the shell microstructure

As a first approach, SEM observations of the periostracum (external shell surface) were made in order to evaluate potential effect of the treatments. These observations were performed at two different places of the shell: (i) the ventral extremity part and (ii) a lower part of the shell. Two individuals per experimental condition (65 days of exposure) were studied. For all the samples, the periostracum was of the same colour (based on visual observations). Under SEM, at the ventral extremity in control condition (29.2°C, 430 *μ*atm), the periostracum did not appear altered (Supplementary Fig. S2A, C; Table 4), in the lower part it displayed a ‘mushy’ aspect with numerous micro-borer galleries (Supplementary Fig. S2B, D). These features were observed in all the other experimental conditions (i.e. 29.2°C, 1212 *μ*atm; 30.7°C, 430 *μ*atm; 30.7°C, 1212 *μ*atm; Fig. S2) and for all the samples studied, which may signify that the treatments did not significantly affect the periostracum.

To strengthen the results above ultrastructural observations of shell fractures were done in two zones: zone 1 corresponding to the shell formed *in situ* (i.e. before the experiment) and zone 2 corresponding to the shell formed during the experimental conditions. In the control condition (i.e. 29.2°C, 430 *μ*atm), after 29 days of exposure, the lamellae formed before the experiment (zone 1) and during the experiment (zone 2) were well cohesive and displayed an elongated shape, a smooth surface and sharp or slightly rounded outlines (Fig. 3A–C). At 29 days, no difference was observed between both zones for all temperature/*p*CO_2_ conditions (example given for 29.2°C, 1212 *μ*atm in Fig. 3D–F; Table 4). From 41 days and after, and for all temperature/*p*CO_2_ experimental conditions, the shell formed before the experiment (zone 1) displayed the same microstructural features like the ones described above (example given for 29.2°C, 430 *μ*atm; 30.7°C, 430 *μ*atm; and 30.7°C, 1212 *μ*atm at 41 days in Fig. 3G, J, M; Table 4). At 41 days, the lamellae observed in the majority of shells formed during the experiment (zone 2), in all temperature/*p*CO_2_ conditions, appeared less cohesive (Fig. 3N, O showing shell formed at 30.7°C, 1212 *μ*atm; Table 4) and some of them additionally displayed a pronounced granular aspect of the surface even in the control condition (Fig. 3H, I and Fig. 3K, L showing shell formed at 29.2°C, 430 *μ*atm and 30.7°C, 430 *μ*atm, respectively; Table 4). These modifications were observed for the majority of shells formed in all temperature/*p*CO_2_ conditions, at 53 and 65 days (Table 4). Two states classifying ultrastructural differences between the shell formed before and during the experiment were defined as follows: (i) ND, no difference observed between zones 1 and 2; and (ii) D, difference observed between zones 1 and 2 meaning that the lamellae were less cohesive in zone 2 compared to zone 1 (see Table 4). In total, 19 samples over 32 displayed differences in lamellae aspect between both zones.

### Effect of time of exposure on oxygen balance, biomineralization and symbiont photophysiology

Time of exposure to thermal and acidification stress was shown to have a significant impact on O_2_ production (*P* = 0.030), photosynthetic yield (*P* = 0.021), symbiont density (*P* = 0.001) and shell growth rate (*P* = 0.007) (Table 2). Post hoc tests showed that the kinetics of thermal or acidification stress varies depending on the physiological parameter measured: O_2_ production and photosynthetic yield were significantly different between 29 and 65 days while symbiont density and shell growth rate were both impacted from 53 days of exposure (Table 3).

**Table 5:** Correlation matrix integrating the different physiological parameters monitored, i.e. O_2_ production and consumption, shell extension rate and symbiont photosynthetic yield and density

	Photosynthetic yield (*n* = 64)	Symbiont density (*n* = 64)	Shell extension rate (*n* = 55)	O_2_ production (*n* = 64)
Symbiont density	0.667^*^			
Shell extension rate	−0.032	0.168		
O_2_ production	0.331^*^	0.492^*^	0.221	
O_2_ consumption	0.163	0.226	0.129	0.629^*^

Correlation was tested using the Pearson method (threshold of *r* = 0.250, α = 0.05) and significant relationships between parameters are indicated by an asterisk (^*^).

### Relationship between physiological parameters

To establish if relationships exist between the various physiological parameters measured, a correlation matrix was generated (Table 5). The photosynthetic yield of symbionts was strongly correlated to their density (*r* = 0.667). O_2_ production was also strongly correlated to symbiont density (*r* = 0.492) and photosynthetic yield (*r* = 0.331). Concerning the circadian functioning of the holobiont, its nocturnal oxygen need was strongly correlated to the diurnal O_2_ production (*r* = 0.629). No significant correlation was found between shell extension rate and other physiological parameters.

## Discussion

In the present study, we investigated the physiological responses of *T. maxima* (i.e. 4-year-old specimens) to temperature and *p*CO_2_ conditions in the French Polynesian lagoons under current warmer season conditions and those predicted for 2100 by the IPCC 2014 (+1.5°C and +800 *μ*atm of CO_2_). Present thermal conditions were stressful enough to induce an acclimatization process characterized by a slight regulation of the symbiont density without the collapse of the photosynthesis. However, the predicted thermal and *p*CO_2_ conditions were much more stressful and induced strong disturbances of the processes linked to the phototrophic symbiosis and the biomineralization.

Indeed, the 30.7°C treatments have significantly impacted the holobiont by inducing a strong bleaching response illustrated by the reduction of its O_2_ production, symbiont density and photosynthetic yield. High *p*CO_2_ (+800 *μ*atm of CO_2_) was shown to alter the symbiont photosynthetic yield and density and affect its biomineralization process by decreasing the shell growth rate.

### No synergetic effect between temperature and pCO_2_ on giant clam and symbiont physiological parameters

Increase in atmospheric CO_2_ impacts both the seawater temperature and pH inducing ocean warming and acidification (Sabine *et al.*, 2004; IPCC, 2014). Therefore, the analysis of the synergetic effect of both stressors on marine bivalve’s physiology is crucial. Watson *et al.* (2012) reported in *T. squamosa* juveniles a decrease of giant clam’s survival rate induced by exposure to high-temperature or high-*p*CO_2_ conditions. The lowest survival rate (i.e. <20%) was observed under the highest *p*CO_2_ condition (+600 *μ*atm of CO_2_) at +1.5°C and +3°C, after 60 days of exposure.

In our study, no synergetic effect was detected for all measured physiological parameters. Moreover, no mortality was observed in all tested temperature/*p*CO_2_ conditions during the whole experiment which leads us to suggest that a temperature of 30.7°C and a *p*CO_2_ of 1212 *μ*atm seem to be non-lethal, at least over 65 days of exposure. However, we cannot exclude that some specimens, especially the bleached individuals, may have died after longer exposure to the experimental conditions.

### Effect of temperature on holobiont oxygen balance, symbiont photophysiology and shell ultrastructure

Temperature is one of the most important parameters driving bivalve physiology (Aldridge *et al.*, 1995; Bougrier *et al.*, 1995; Watson *et al.*, 2012; Gazeau *et al.*, 2013; Le Moullac *et al.*, 2016a; Latchère *et al.*, 2017). However, since giant clams live in symbiosis with photosynthetic symbionts (Klumpp *et al.*, 1992; Klumpp and Griffiths, 1994; Soo and Todd, 2014), the comparison at the metabolic level with non-symbiotic bivalves can be misleading. Discussion on elevated temperature effect on the holobiont oxygen balance should therefore integrate the effect on both giant clam and its symbionts (Jones and Hoegh-Guldberg, 2001). Even though physiological and molecular responses to thermal stress may differ, the comparison with scleractinian corals, which are also symbiotic and calcifying organisms, makes sense to better understand the impact of temperature on the holobiont physiology.

We observed that high temperature (+1.5°C) significantly reduced the holobiont O_2_ production, the density and the photosynthetic yield of symbionts from 29 days of exposure. Additionally, partial or total bleaching was observed for the majority of individuals exposed to 30.7°C (at both ambient and high *p*CO_2_, see Supplementary Fig. S3). This suggests that thermal stress has a significant impact on the holobiont photophysiology. The reduction of the symbiont photosynthetic yield could reflect photoinhibition. Photoinhibition was previously linked to the degradation of the D1 protein of the reaction center of the PSII altering the photosynthetic apparatus functioning in symbiotic corals and sea anemone subjected to thermal stress (Warner *et al.*, 1999; Smith *et al.*, 2005; Richier *et al.*, 2006; Ferrier-Pagès *et al.*, 2007). Decrease of photosynthetic activity reduced the symbiont O_2_ production and consequently, impacted the holobiont O_2_ production. Comparable results were obtained from a 24-h experiment showing that an increase of 3°C affected the oxygen production of *T. gigas* and *T. derasa* (Blidberg *et al.*, 2000). Photoinhibition led to the production of ROS by symbionts, which are known to pass through cellular membranes, cause oxidative damages (Lesser *et al.*, 1990; Lesser, 1996; Downs *et al.*, 2002) and impact the PSII (Richter *et al.*, 1990). In corals, Downs *et al.* (2002) showed that high levels of oxidative damage associated with coral bleaching. Indeed, ROS such as hydrogen peroxide (H_2_O_2_) may play a role in signalling molecule activating the symbiosis dissociation (Fitt *et al.*, 2001; Smith *et al.*, 2005; Perez and Weis, 2006). Expulsion of symbionts by the host may be a strategy to limit oxidative stress and damage to ultimately survive environmental stress (Downs *et al.*, 2002; Perez and Weis, 2006). Two recent works support this hypothesis. Firstly, Zhou *et al.* (2019) related a decrease in symbiont density in response to an excess of oxidative stress in thermally stressed *T. crocea*. Secondly, Dubousquet *et al.* (2016) showed that *T. maxima* overexpressed genes encoding ROS scavengers in response to thermal stress. Interestingly, the cellular and molecular mechanisms enhanced before the bleaching response (e.g. the loose of the symbionts) seem similar between giant clams and corals and conduct to the same phenomenon of symbiosis dissociation. However, since giant clams and corals display an extra- and an intra-cellular symbiosis, respectively, the mechanisms of symbiosis dissociation may differ. This constitutes an interesting example of evolutionary convergence of a stress response in two very distant organisms. In this context, it would be interesting to test if the bleaching response in giant clam can be adaptive as it was proposed for corals (Fautin and Buddemeier, 2004) and if variations in giant clam thermo-tolerance are correlated to the composition of the symbiotic population (Baker, 2003).

Concerning the effect of temperature on the ultrastructure of the crossed-lamellar structure, the lamellae formed in all experimental conditions in the first 29 days were well cohesive with elongated shape and a slight granular aspect, which is consistent with the description made by Faylona *et al.* (2011) in *T. maxima*, Belda *et al.* (1993) in *T. gigas* and Agbaje *et al.* (2017) in *T. derasa*. However, after 29 days, the lamellae observed in the majority of the shells formed in both temperature conditions (whatever the *p*CO_2_ condition) appeared to be less cohesive with pronounced granular aspect. We suggest that these features are due to a lack of organic matrix between the lamellae (i.e. inter-lamellar organic matrix) and embedding the nano-grains forming the lamellae in the crossed-lamellar structure. Indeed, the biomineralization of calcium carbonate structures involves the transport of ions to the mineralization site and the synthesis of macromolecules referred to as ‘organic matrix’ (Allemand *et al.*, 2004). The organic matrix, consisting of 0.9 wt% and 1.83 wt% in *T. derasa* and *T. gigas* shell, respectively (Agbaje *et al.*, 2017, 2019), is mainly composed of macromolecules commonly found in molluscan shell organic matrix such as lipids, polysaccharides and proteins displaying various levels of glycosylation (Marin *et al.* 2012; Agbaje *et al.*, 2017). The formation of these macromolecules may be energetically costly for molluscs such as marine gastropods (Palmer, 1992). We suggest that in our tested experimental temperature, while oxygen balance is significantly altered, less energy may be available and allocated to the synthesis of macromolecules involved in biomineralization, which may explain the lack of organic matrix embedded into the giant clam shell.

### Effect of pCO_2_ on giant clam biomineralization and symbiont photophysiology

Results of the present study have indicated that in *T. maxima* high-*p*CO_2_ condition (+800 *μ*atm) induced a significant decrease of the shell extension rate. This observation is in accordance with those obtained for giant clams in three different experiments using various CO_2_ enrichment levels and exposure durations. Watson (2015) has exposed juveniles *T. squamosa* to +250 and +550 *μ*atm of CO_2_ for 8 weeks under various light levels. Under mid-light level, the author has reported a reduced survival and growth while under high-light levels the growth only was affected. Kurihara and Shikota (2018) also reported a negative effect of *p*CO_2_ on shell growth (i.e. shell height) in juveniles *T. crocea* when exposed to +600 *μ*atm and +1600 *μ*atm for 4 weeks. Toonen *et al.* (2012) demonstrated that young specimens of *T. maxima* and *T. squamosa* had lower shell growth rates, compared to those reported in the literature under natural *p*CO_2_/pH conditions, when kept 1 year in +350 to +1000 *μ*atm *p*CO_2_ conditions.

As mentioned above, high-*p*CO_2_ condition (+800 *μ*atm) affected giant clam’s shell growth rate and also photosynthetic yield and density of symbionts. Negative effect of *p*CO_2_ on the shell growth rate may be linked to two non-exclusive hypotheses: (i) the physiological adjustment needed to thrive with a seawater at 1212 *μ*atm of CO_2_ instead of 430 *μ*atm and/or (ii) an alteration of symbiont photophysiology leading to a potential reduction of the ‘light-enhanced calcification’ (LEC) phenomenon. Concerning the former, the increase of CO_2_ dissolution in the water column leads to the modification of the carbonate chemistry equilibrium and to the increase of H^+^ concentration (Cyronak *et al.*, 2016). These strong environmental changes affect the whole organism’s homeostasis, but more particularly physiological functions, such as biomineralization, where the control of carbonate concentration and pH are the most essential. To form their mineralized structures, calcifying organisms modify the carbonate composition at the mineralization site to promote CaCO_3_ precipitation. These modifications are performed by several enzymatic reactions, including the removal of H^+^ to locally increase the pH at the mineralization site for maintaining chemical conditions to enhance CaCO_3_ precipitation (Allemand *et al.*, 2011; Taylor *et al.*, 2012). Corals are known to regulate the pH at their mineralization site (Venn *et al.*, 2013; Holcomb *et al.*, 2014) but a decrease of coral calcification was also linked to a decline in pH in the calcifying fluid (Ries, 2011; McCulloch *et al.*, 2012). The maintenance of biomineralization under these non-optimal environmental conditions are therefore energetically costly, which may result in a general decrease of growth (Vidal-Dupiol *et al.*, 2013). In our case, we suggest that giant clam *T. maxima* exposed to high *p*CO_2_ may allocate more energy to maintain a proper pH of the extrapallial fluid for nucleation and deposition of aragonite. Regarding the LEC (Vandermeulen *et al.*, 1972), this phenomenon observed in Symbiodiniaceae-host symbiosis has been extensively described in corals and represents the capacity of symbionts to stimulate the host calcification (Allemand *et al.*, 2004). The symbionts stimulate the host metabolism and calcification by providing energy resources and/or O_2_ (Chalker and Taylor, 1975). Moreover, symbionts may promote the aragonite precipitation by providing inorganic carbon, nitrogen and phosphorus and by synthetizing molecules used as precursor for the synthesis of skeletal organic matrix (Pearse and Muscatine, 1971; Cuif *et al.*, 1999; Furla *et al.*, 2000; Muscatine *et al.*, 2005). They also facilitate CaCO_3_ precipitation by influencing the dissolved inorganic carbon (DIC) equilibrium by removing the CO_2_ via photosynthesis (Goreau, 1959). Such phenomenon has been also described in giant clams (Ip *et al.*, 2006; Ip *et al.*, 2017). In *T. squamosa*, LEC increased the pH and reduced the ammonia concentration at the interface between the inner mantle and the shell in the extrapallial fluid, where the biomineralization occurs (Ip *et al.*, 2006). Recently, Chew *et al.* (2019) reported a light-enhanced expression of carbonic anhydrase, i.e. CA4-like, in the inner mantle of *T. squamosa* and suggested that this enzyme is involved in giant clam biomineralization by catalyzing the conversion of HCO_3_^−^ to CO_2_. In this context, an altered photophysiology of the symbionts can rationally alter LEC and consequently results in a decrease of the shell growth rate. Finally, one can suggest that under acidification stress, giant clam may reduce some physiological functions such as biomineralization and allocate more energy to essential functions for its survival. In our study, temperature also altered photophysiology and holobiont O_2_ production, but did not significantly affect shell growth rate. Therefore, the most plausible hypothesis explaining the negative effect of *p*CO_2_ on shell growth rate may be related to the low aragonite saturation state at high *p*CO_2_.

Concerning the effect of *p*CO_2_ on the shell microstructural integrity, in the temperate bivalve *Mytilus edulis*, an exposure to +150, +350 and +600 *μ*atm of CO_2_ for 6 months induced disorientation in the shell of the newly formed calcite crystals of the prismatic layer (Fitzer *et al.*, 2014). In the giant clam *T. maxima*, high *p*CO_2_ had no significant effect on the integrity of the aragonitic lamellae of the crossed-lamellar layer during the first 29 days of exposure. From 41 days of exposure, its potential impact remains unresolved as differences were noticed in the shells formed under future high-temperature, high-*p*CO_2_ and even under today’s temperature/*p*CO_2_ conditions. The fact that differences were reported for shells formed in all experimental conditions from 41 days suggests that they may be due to a long-term exposure to 29.2°C and 30.7°C.

## Conclusion

This study enables the evaluation of *T. maxima*’s physiological responses to realistic temperature and *p*CO_2_ predicted at the end of this century. We demonstrated that high temperature mimicking temperature encountered during the warmer months of the year has a significant negative impact on symbiont densities and photosynthetic capacities. This negative impact on the symbiont physiology induces a decrease in the net holobiont O_2_ production.. Therefore, by influencing symbiont physiology, the temperature may affect the energetic needs of the giant clam host. The high *p*CO_2_ has a negative impact on shell growth rate, symbiont densities and photosynthetic capacities. Shell microstructure is not affected by temperature nor by the *p*CO_2_ in the first 29 days of exposure. However, for all temperature/*p*CO_2_ conditions, a longer exposure (≥41 days) modified the shell ultrastructure. These observations support our hypothesis that 29.2°C is a temperature that already affects giant clam metabolism, at least over a long-term exposure. However, no synergetic effect was found between temperature and *p*CO_2_ parameters. All these observations suggest that temperature and *p*CO_2_ influence different physiological functions and that giant clam populations may dramatically suffer from the temperature and *p*CO_2_ conditions predicted for the next decades. This is especially true for the temperature since the populations of giant clams (wild and farmed) of the Tuamotu Archipelago are already confronted with temperatures of ~29.2°C every year. Additionally, the threshold of 30.7°C applied in our study and that will be encountered annually in the future corresponds to what is measured currently during abnormally warm years (Fig S1). During the heatwave event in 2016, 80% of the farmed clam populations have bleached resulting in a high level of mortality (personal communication). To complement these results on the effects of temperature on the giant clams, it is now essential to conduct integrative analyses that will take into account the acclimatization and adaptive potential of the whole holobiont. The tools of transcriptomic, genomic and epigenomic in association with ecologically relevant experiments both at the individual and population levels will be particularly relevant to address these questions (Torda *et al.*, 2017). These will allow a better understanding of the fundamental physiological processes of the holobiont and its response to future changes. Results from these studies may help in adapting local policies and management to maintain sustainability of giant clam populations and their exploitation, especially in the Eastern Tuamotu islands where bleaching events have been observed at an increasing and alarming rate.

## Funding

This work was supported by the Ifremer Institution (Politique de site program, GECO project) and the Université de la Polynésie française (MAPIKO and CLAMS projects).

## Conflicts of interest

The authors declare no financial and personal conflict of interest.

## Supplementary Material

suppl_data_coab041
