# Supplementary figures and images for: Effects of elevated temperature and pCO2 on the respiration, biomineralization and photophysiology of the giant clam Tridacna maxima
Source: Conserv Physiol. 2021 Jun 16;9(1):coab041. doi: 10.1093/conphys/coab041 (PMC8208665; doi:10.1093/conphys/coab041)

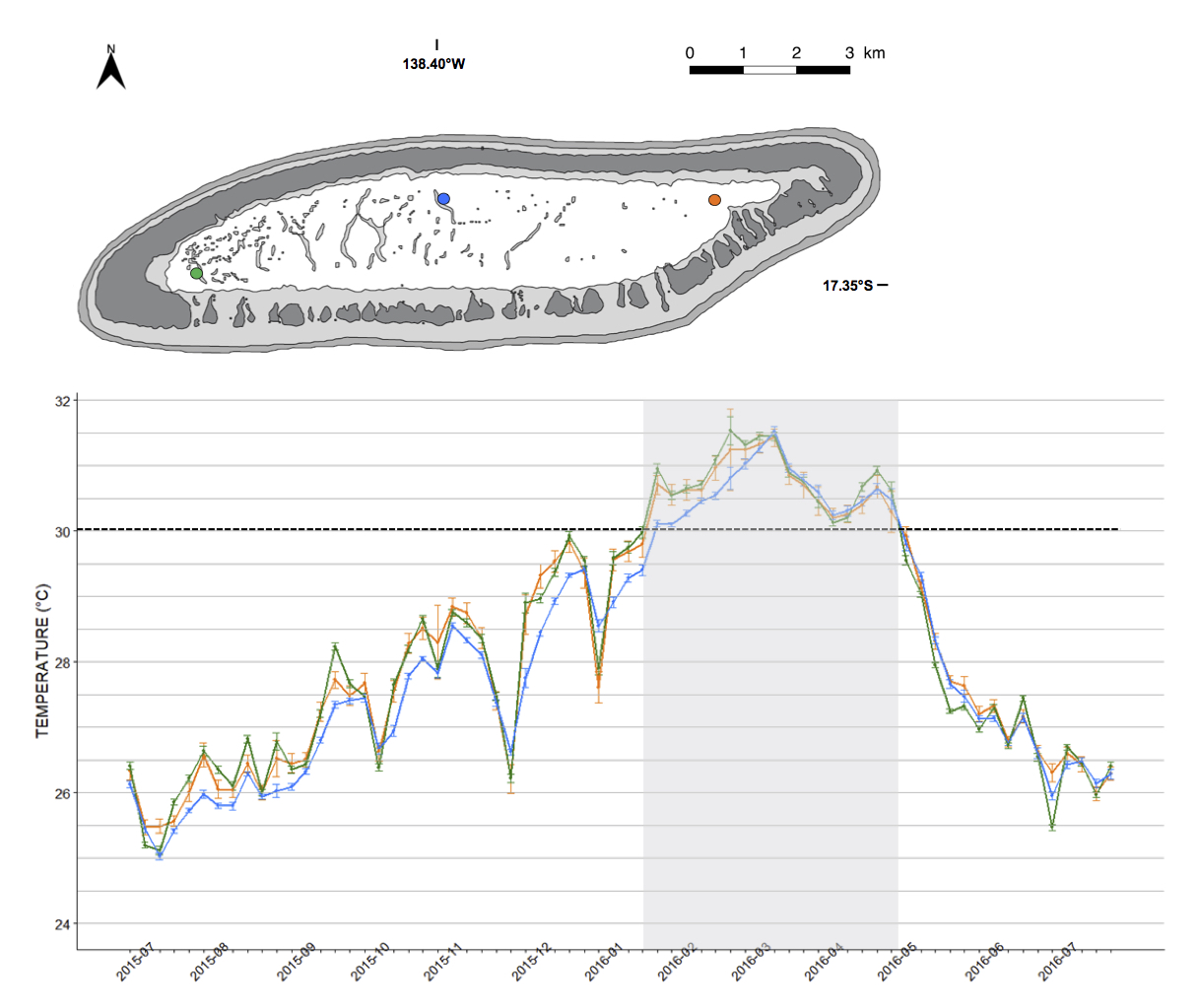

Supplement: suppl_data_coab041 [file suppl_data_coab041.zip › Supplementary Figure S1.tiff]

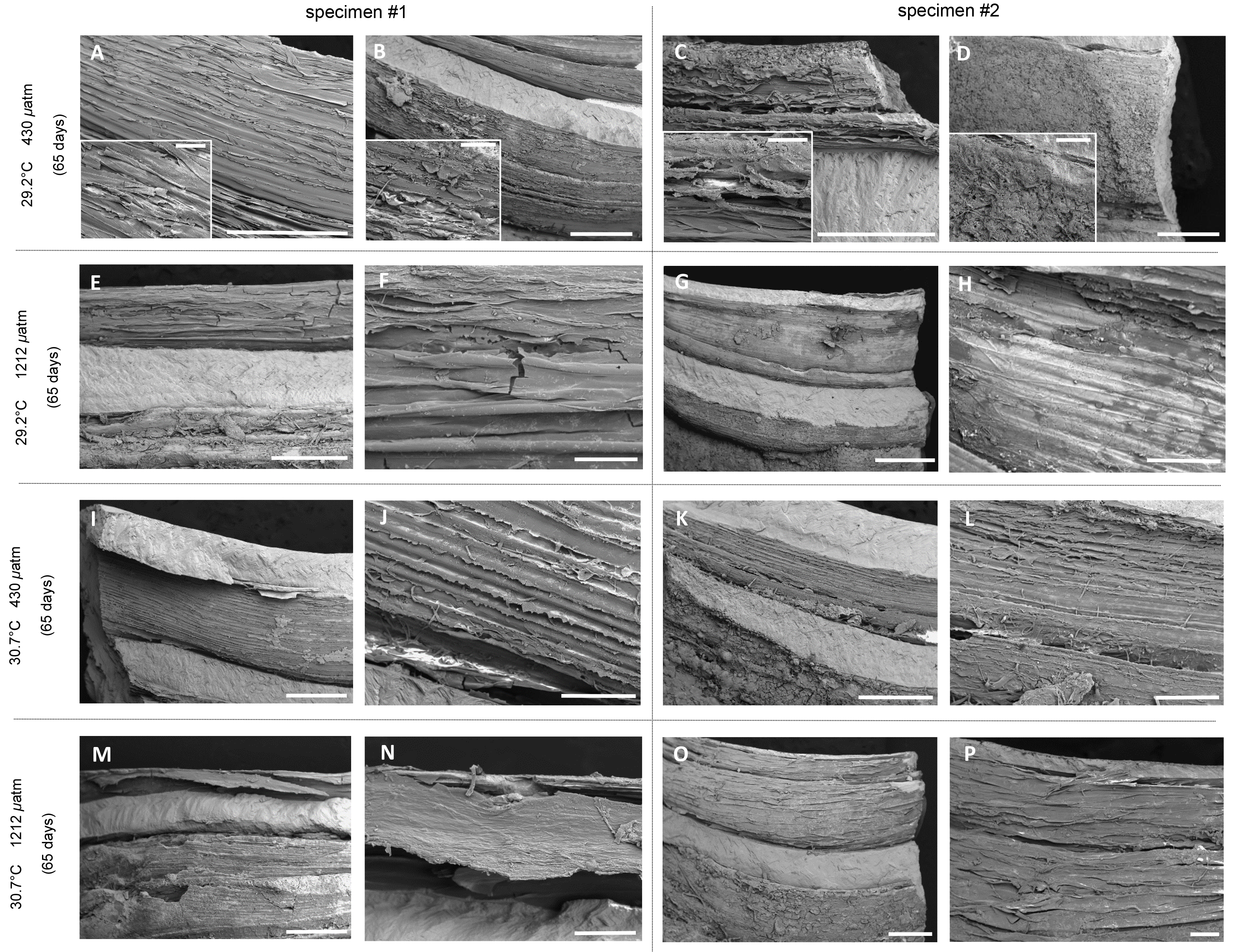

Supplement: suppl_data_coab041 [file suppl_data_coab041.zip › Supplementary Figure S2.jpg]

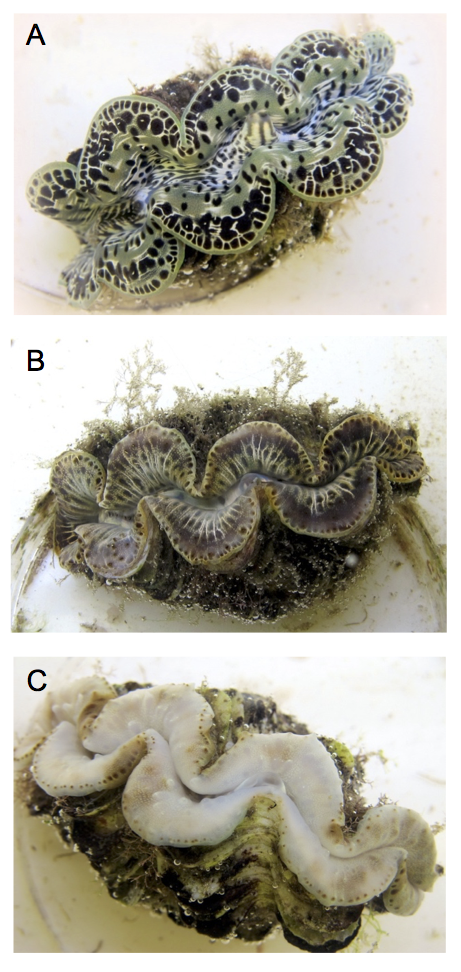

Supplement: suppl_data_coab041 [file suppl_data_coab041.zip › Supplementary Figure S3.tiff]
